# Changes in Competitors, Stress Tolerators, and Ruderals (CSR) Ecological Strategies after the Introduction of Shrubs and Trees in Disturbed Semiarid Steppe Grasslands in Hulunbuir, Inner Mongolia

**DOI:** 10.3390/biology12121479

**Published:** 2023-11-30

**Authors:** Eui-Joo Kim, Seung-Hyuk Lee, Se-Hee Kim, Jae-Hoon Park, Young-Han You

**Affiliations:** 1 Department of Biological Sciences, Kongju National University, Gongju 32588, Republic of Korea; euijoo@kongju.ac.kr (E.-J.K.); ksh41631@smail.kongju.ac.kr (S.-H.K.); kn5314@smail.kongju.ac.kr (J.-H.P.); 2 Garden Promotion Department, Korea Arboreta and Gardens Institute, Sejong-si 30129, Republic of Korea; sensus@koagi.or.kr

**Keywords:** plantation, restoration, functional group diversity, life-form, succession

## Abstract

**Simple Summary:**

Grasslands cover one-third of the world’s land area and play a significant role in agriculture and ecological security. However, due to recent changes in precipitation patterns and land use, the coverage of grasslands is decreasing. The Hulunbuir steppe, one of the four major grasslands in the world, is also undergoing desertification caused by climate change and anthropogenic disturbances. To characterize the eco-functional diversity of plants in recently restored sites after the introduction of woody plants in natural grasslands, the competitor, stress tolerator, and ruderal (CSR) ecological strategies of plants were analyzed. As a result, the CSR ecological strategy in the temperate typical steppe was CSR and that in the woodland steppe was S/SR. The restored sites differed depending on the life-form of the introduced woody plants. The CSR ecological strategies observed over time in a restored site after the introduction of woody plants varied depending on the life-form of the introduced woody plants. This suggests the importance of selecting the life-form of woody plants for the success of ecological restoration in damaged grasslands.

**Abstract:**

To reveal the changes in the life history characteristics of grassland plants due to vegetation restoration, plant species and communities were analyzed for their competitor, stress tolerator, and ruderal (CSR) ecological strategies after the introduction of woody plants in the damaged steppe grassland and were compared with those in reference sites in Hulunbuir, Inner Mongolia. As a result, it was found that the introduction of the woody plants (*Corethrodeneron fruticosum*, *Caragana microphylla*, *Populus canadensis*, and *Pinus sylvestris* var. *mongolica*) into the damaged land greatly increased the plant species diversity and CSR eco-functional diversity as the succession progressed. The plant strategies of the temperate typical steppe (TTS) and woodland steppe (WS) in this Asian steppe are CSR and S/SR, respectively, which means that the plants are adapted to disturbances or stress. As the restoration time elapsed in the damaged lands exhibiting (R/CR) (*Corispermum hyssopifolium*), the ecological strategies were predicted to change in two ways: (1) →R/CSR (*Cynanchum thesioides*, *Astragalus laxmannii*, etc.) → CSR in places (TSS) (*Galium verum* var. *asiaticum*, *Saussurea japonica*, etc.) where only shrubs were introduced, and (2) → S/SR (*Allium mongolicum*, *Ulmus pumila*, etc.) → S/SR in sites (WS) (*Ulmus pumila*, *Thalictrum squarrosum*, etc.) where trees and shrubs were planted simultaneously. The results mean that the driving force that causes succession in the restoration of temperate grasslands is determined by the life-form (trees/shrubs) of the introduced woody plants. This means that for the restoration of these grasslands to be successful, it is necessary to introduce woody tree species at an early stage.

## 1. Introduction

Grassland ecosystems are the most widely distributed terrestrial biomes in the world and are classified into steppes, prairies, pampas, savannas, and tundra, especially based on average annual temperatures and precipitation [1,2]. Grasslands provide important ecosystem services, such as providing protective cover for soil, primary production, maintenance of the diversity of ecosystems, and biodiversity [3,4,5,6,7]. However, most grasslands are threatened by continuous overgrazing, the conversion of farmlands, deforestation, and indiscriminate reclamation, which has led to a severe decline in grasslands [8,9,10]. At the same time, grassland ecosystems are being devastated by decreases in precipitation due to climate change, continual forest fires and drought, and the deterioration of land conditions [8,11]. To preserve grassland ecosystems, which are degraded as environmental changes such as changes in climate and physical environments progress, it is urgently needed to clarify the necessity of protection and take measures to realize restoration for the harmonious development of humans and nature [12,13].

The Hulunbuir temperate steppe in the northwest of Hulunbuir in Asia is one of the world’s four largest grasslands, accounting for 22% of the total area of China [5,14,15,16]. The Hulunbuir steppe hosts one of the types of vegetation that are sensitive to environmental changes such as climate change; its vegetation has great significance in terms of ecosystem services because of its role as a national green ecological barrier and in providing ecological security [7,17]. However, in addition to recent changes in precipitation patterns due to climate change, the land is severely damaged due to the conversion of land use from grasslands to farmlands or from woodland steppe to farms and rapid increases in the number of livestock [18]. Consequently, the area of the grasslands is rapidly decreasing [15], and desertification is progressing due to land degradation [19,20]. To preserve the Hulunbuir steppe, the Chinese government started a project to prevent desertification and yellow dust damage in the 2000s and to restore the grassland ecosystem together with residents, and it has been making great efforts such as with monitoring studies and tree planting until recently [21].

The life history strategy of plants is greatly influenced by environmental factors [22,23], and the interaction between plants and environmental factors affects ecosystem functions [24]. Plants growing in a spatial and temporal microenvironment have similar life history patterns [25], and the characteristics of ecological functional groups among constituent species in the same place can be known [26]. Grime expanded the concepts of r and K selection to include the patterns of species found in different types of habitats and introduced the C (competitor), S (stress tolerator), and R (ruderal) ecological strategy concept [27]. Among the methods of evaluating the life history strategies of plants according to environmental changes, the analysis of the CSR ecological strategy is useful for evaluating plant ecological strategies [27] because it enables an understanding of the functional characteristics of constituent species under similar environmental factors [28].

Therefore, analysis of the CSR ecological strategy should be an effective method to use when it is necessary to explain the future state of the grassland ecosystem by revealing the functional characteristics of the community’s constituent species, which are gradually declining due to disturbances by climatic, environmental, and physical factors. In addition, since the effect of the changing environment on plant characteristics can be demonstrated with CSR ecological strategies [29], they are judged to be useful as a means of evaluating the effectiveness of restoration. However, studies related to the CSR ecological strategies of grassland ecosystems are rare globally, and no study has been conducted with the Hulunbuir temperate steppe.

Therefore, the purpose of this study is to investigate the CSR ecological strategies of plant species and communities in restored sites where vegetation was introduced into degraded grasslands, desertification areas, and well-conserved reference site grasslands (typical grasslands and woodland steppe) and to compare and analyze the characteristics of the restored sites and the reference sites in an attempt to find the life historical characteristics of plants in temperate grasslands in order to preserve the Hulunbuir temperate steppe.

## 2. Materials and Methods

### 2.1. Overview of the Study Site

**Figure 1:**
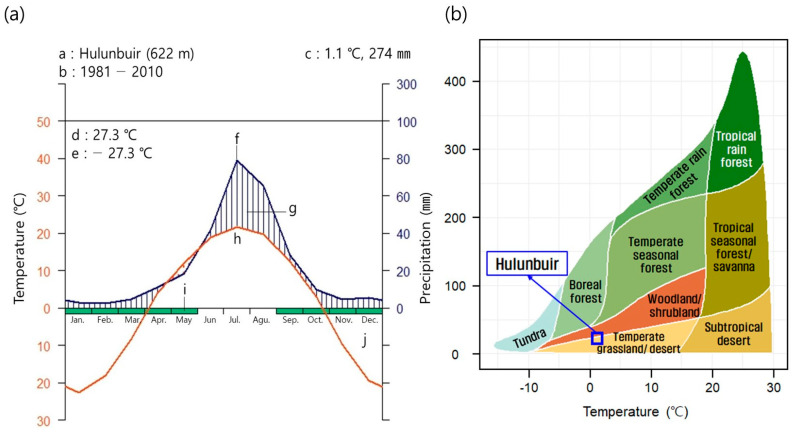
Diagram of climate from 1981 to 2010 (**a**) and biome classification according to Whittaker (1962) (**b**) in Hulunbuir. a: location name and altitude, b: the period of observation, c: mean temperature and annual mean precipitation, d: maximum temperature of warmest month, e: minimum temperature of coldest month, f: mean monthly temperature, g: humid period, h: mean monthly precipitation, i: a month in which the average daily minimum temperature is 0 °C or lower, j: the dry season in the left figure (**a**). In the right figure (**b**), the blue square means the corresponding biome in Hulunbuir.

This study was conducted in the steppe (N 48°04′~48°27′, E 119°18′ to 118°17′) located in Ganzhuer, Hulunbuir in the northeastern part of the Inner Mongolian Autonomous Region, China where trees were planted from 2014 to 2018 as part of the prevention of desertification. In Hulunbuir, a temperate typical steppe is developed by Siberian high pressure caused by western Pacific subtropical high pressure and the East Asian summer monsoon [30]. Walter et al. created a climatic map [31] for the climate of the Hulunbuir steppe (1981–2010) using data from the China Meteorological Observation Network [32] measured by the National Meteorological Observatory of Inner Mongolia (Figure 1a). The average annual temperature was 1.1 °C and the annual precipitation was 422 mm (Figure 1a). In Whittaker biomes [33,34], Hulunbuir is between temperate grassland/desert and woodland/shrubland (Figure 1b). In addition, in the climate classification standard of Koppen–Geiger, Hulunbuir corresponded to BSh (steppe climate and hot arid) and thus was classified as a temperate steppe [35].

**Figure 2:**
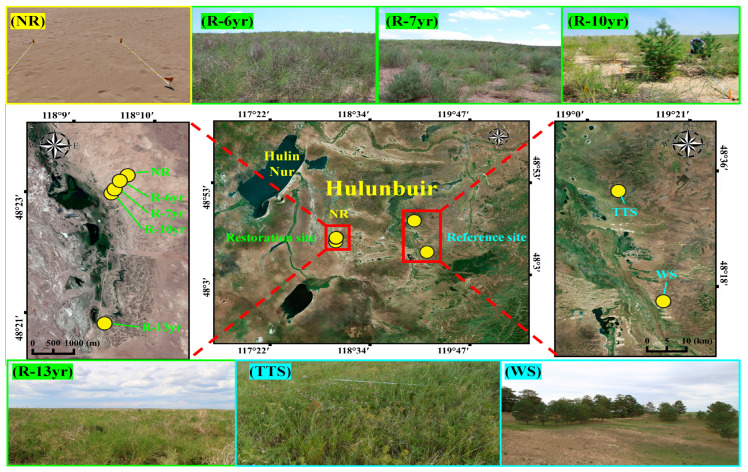
The four photographs at the top are landscapes (from right to left) of restored sites (R-6yr, R-7yr, R-10yr) including the unrestored site (NR). In the center photographs, the one on the left shows the location of the unrestored site and the restoration point, the one in the center shows the location of all research stations in the Hulunbuir area, and the one on the right shows the location of the reference ecosystem. From the right to the left at the bottom, the photographs show the restored area (R-10yr), temperate typical steppe (TTS), and woodland steppe (WS). The red boxes indicate the location of the research sites, and the dotted line is the direction for the enlarged photograph of the location of the research site. Abbreviation explanation: R-(number/yr) the number of years elapsed since restoration, TTS means temperate typical steppe, WS means woodland steppe, and NR means non-restoration.

This research area is a place where the Forestry Bureau of China has carried out a restoration project for grassland devastation and desertification since 2005. Since 2012, Chinese officials have conducted research on monitoring after restoration jointly with the Korea–China–Japan Yellow Dust Joint Research Group Working Group Ⅱ (WG Ⅱ). The restored sites were classified according to the time elapsed after restoration by planting, and the sites comprise a total of four points in the sand dune terrain (Table 1). In the case of the point where vegetation was restored in 2005 (R-13yr), 13 years of restoration time had elapsed, and in the case of the point where vegetation was restored in 2008 (R-10yr), about 10 years of restoration time had passed. In addition, in the case of the points where vegetation was restored in 2011 and 2012 (R-7yr and R-6yr), 7 years and 8 years of restoration times had passed, respectively. The reference sites were the non-restoration point (NR) exposed to a floating dune where restoration was not performed, the temperate typical steppe (TTS), and the woodland steppe (WS), which are the terrain of fixed dunes (Figure 2). The TTS is a natural grassland ecosystem protected in Hulunbuir, China, and is called Yi lite. TTS is protected with pillars and wire netting and WS is a *P. sylvestris* var. *mongolica* community, a small-scale remnant vegetation, which is remaining in the region between the forest of Hulunbuir called Nanhui and the grasslands (Figure 2).

The trees selected and planted for vegetation restoration were *P. sylvestris* var. *mongolica* and *Populus canadensis*, which are tree plants that grow wild in Hulunbuir, and *Caragana microphylla* and *Corethrodeneron fruticosum*, which are shrub plants. As for the vegetation restoration method, seed sowing or tree planting was applied (Table 1). In R-6yr, seed sowing and shrub planting were applied simultaneously, and in R-7yr, R-10yr, and R-13yr, trees and shrubs were planted simultaneously (Table 1). The dominant species in individual study sites was *C. fruticosum* in NR and R-6yr, *P. canadensis* in R-7yr and *C. fruticosum* in the underlayer. Also, *P. sylvestris* was dominant in R-10yr, and *C. microphylla* was dominant in the underlayer. In addition, in the TTS, *Galium verum* var. *asiaticum,* which is an herb, was dominant, and in the WS, *P. sylvestris* var. *mongolica* was dominant (Table 1).

To fix the sand in all restored sites, checkerboard barriers were made using straw and reeds to prevent sand movement. In addition, 1.5 m high barbed-wire fences were installed around all the restored sites to protect vegetation from livestock grazing [36].

### 2.2. CSR Classification and Functional Group Diversity Comparison

To analyze the types of CSR plant functions by species of the vascular plants that appeared in the Hulunbuir research site, a total of seven traits: canopy height, leaf dry weight, dry matter content, specific leaf area, lateral spread, flowering start, and flowering period, which are predictor variables, were measured [37]. As for canopy height (cm), the height of the above-ground part of the target plant was recorded in centimeters using a ruler or tape measure and converted to millimeters for later analysis. As for leaf dry weight (g), the fresh weights of leaves were measured, the leaves were dried in a 65 °C natural circulation dryer for 48 h, the dried leaves were weighed, and the average value was used. As for dry matter content (%), the ratio of the leaf dry weight to the weight of the leaf saturated with water was obtained and converted to a percentage. The specific leaf area was calculated by dividing the leaf area by the dry weight of the leaf. Lateral spread means the spreading ability of plants, and the distance between creeping stems or ramets was measured, or the life-form was applied in some cases. The flowering start and flowering period were observed firsthand in the field or referred to the *Illustrated Book of Northern Grassland Plants in China* [38,39] or *Flora of China* in English [40].

The analysis of CSR ecological strategies of plants used three CSR model systems and the global plant CSR analysis tool (StrateFy) provided by Pierce et al. [27], the C-S-R Signature calculator provided by the Unit of Comparative Plant Ecology (UCPE) Sheffield of Hunt et al. [26], and the CSR classification tools of Pierce et al. [41]. However, since StrateFy, the newest analysis method of Pierce et al. [27], which has the largest number of sample data and can be applied to the flora of the world, is efficient and was used as the standard, and other models were used as reference data. The C-S-R ecological strategy of each plant was identified with the values derived by inputting values suitable for each trait item among the measured values of plant traits using the above tools. According to the CSR model, C, S, and R, which are three basic strategies, are the primary types, the major secondary strategies are classified into CR, SR, CS, and CSR, and as for the tertiary types, the primary and secondary strategies are mixed and classified, resulting in 19 CSR functional types [37]. It is unfortunate that the analysis of the C-S-R ecological strategy of plants in this study was conducted on species that only appear in certain seasons rather than plants that appear in all seasons in the study area. For species distributed in this study site for which data on other plant characteristics were not obtained, the sample data of the CSR analysis model was used as a reference. Nevertheless, it is meaningful in that the season in which the dominant species in the study area were most active and most abundant was selected, and the C-S-R ecological strategies of plants were analyzed.

To compare vegetation through the arrangement of ecological strategies of communities and track the movements of and changes in the ecological strategies of communities, values were input into the CSR comparator provided by UCPE Sheffield [26] based on the CSR ecological strategy of each plant species, and the derived CSR ecological strategies were identified. The distances between habitats were calculated with Cartesian distances, and the closer the Cartesian distance is to 1, the longer the distance, and the closer the Cartesian distance is to 0, the shorter the distance [26]. Based on the differences in distance, the strategy properties of the communities were identified and compared by community, and the CSR ecological strategy direction of the restored site was predicted. Grime’s CSR triangular system graph was created with the C-S-R ecological strategy scores using SigmaPlot version 15.0 (Systat Software, Chicago, IL, USA), which is a statistical analysis program. To compare eco-functional diversity, the Shannon–Wiener diversity index (H′) [42] was calculated, and the formula used at this time was H′ = −∑ (Pn ln Pn). Pn is the relative abundance, which is the ratio of the nth species within a community, and it means the value obtained by dividing the number of individuals of each taxon by the total number of individuals.

### 2.3. Plant Species Composition and Diversity

Three permanent quadrats (4 m^2^) were installed at each research site, the names of plant species appearing in the permanent quadrats were recorded every year from 2014 to 2018, and the number of species and the number of individuals were investigated. The Shannon–Wiener diversity index (H′) [42] was calculated to compare plant species diversity at NR, restored sites (R-13yr, R-10yr, R-7yr, R-6yr) and reference sites (TTS and WS) for 5 years. The total number of species and the average number of species of the appearing plants were compared, and TTS and WS were expressed as one community. To show clear results, the numbers of species that appeared according to the research sites were expressed as log. Also, plant species were identified referring to the *Illustrated Book of Northern Grassland Plants in China* [38,39] or *Flora of China* in English [32]

## 3. Results

### 3.1. Plant CSR Ecological Strategies

**Figure 3:**
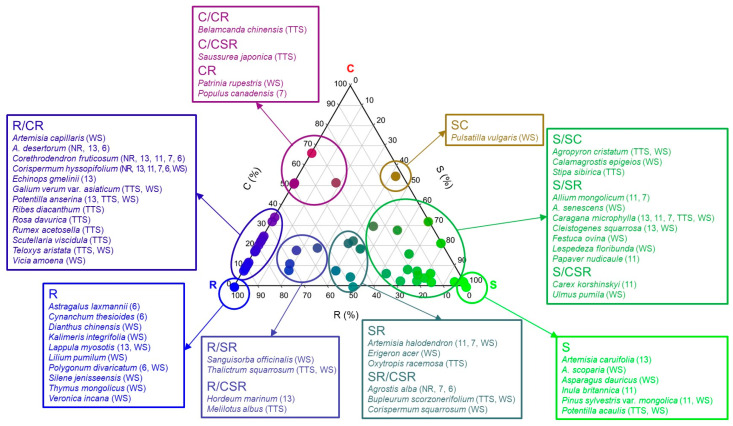
Ternary plot showing relative proportions (%) of C, S and R selection for 56 taxa measured at the research site in Hulunbuir. Species corresponding to the CSR strategy class proposed by Grime (2001) are marked with the species name in the box. Ticks on the axes indicate the matching gridlines inside the triangle. In trigonometry, the classification of C-S-R types is expressed with the colors red (C-selected), green (S-selected), and blue (R-selected), and the color of the point is the RGB value provided by the StrateFy CSR analysis tool. Abbreviation explanation: The numbers inside parentheses represent the elapsed years since the restoration. TTS means temperate typical steppe, WS means woodland steppe, and NR means Non-restoration.

Among the 19 types of plant CSR ecological strategies for 56 taxa, 14 were found in the Hulunbuir research site (Figure 3 and Figure S1). Among them, the R/CR type showed the highest ratio at 48.2% with 27 taxa, which was followed by the S/SR type with 14 taxa (25.0%), R type with 12 taxa (21.4%), S type with eight taxa (14.3%), and SR/CSR type with six taxa (10.7%), and other types showed ratios not higher than 10% with one to three species (Figure 3). Among the CSR types, five (C, C/SC, CR/CSR, SC/SCR, and CSR) were not found in this grassland.

All 61 ecological strategies were arranged in one triangle diagram, and according to the results, the tertiary (R/CR. S/SR) and primary (S, R) types had large distributions among the primary, secondary, and tertiary C-S-R types (Figure 3). All the ecological strategies of the major restored tree species planted in the damaged land were different from each other, as they were S type in the case of *P. sylvestris* var. *mongolica*, CR type in the case of *P. canadensis*, R/CR type in the case of *C. microphylla*, and S/SR type in the case of *C. fruticosum* (Figure 3).

### 3.2. Changes in the Communities’ CSR Ecological Strategies

**Figure 4:**
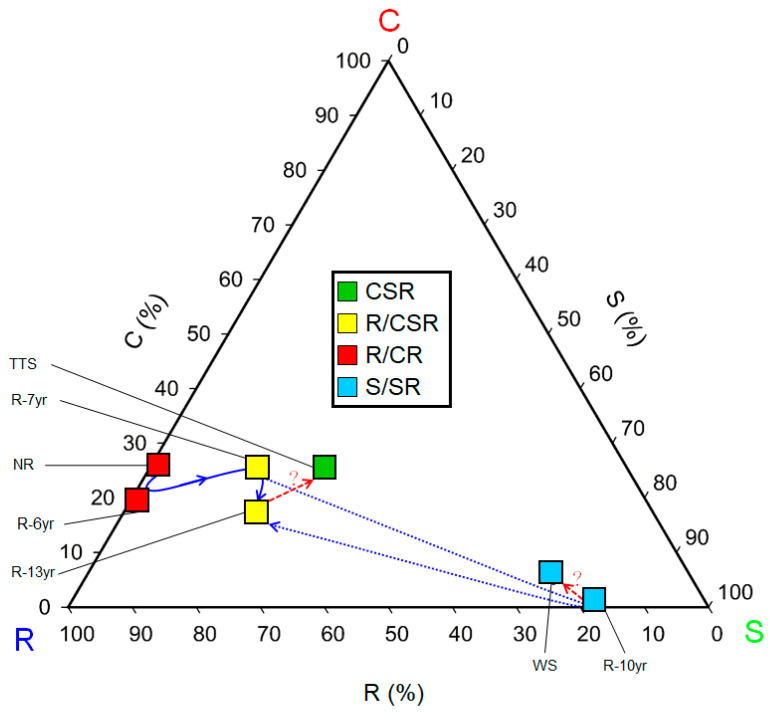
Trinomial plot showing the relative percentages of C, S, and R selection for communities at the study site in Hulunbuir. The boxes in the triangle diagram are arranged in relative proportions of each institute’s community CSR ecological strategy, and the color represents the community CSR ecological strategy, as shown in the legend at the top. The solid blue arrows indicate the direction from dry land to restored land and reference ecosystems. The arrows on the blue dotted line indicate the projections of transitions in the order of restoration elapsed time. The red dotted line and question marks represent subliminal predictions moving forward to the reference ecosystem. The scales of the axes of the triangular degree indicate the coincident grid lines inside the triangle. The classification of C-S-R types in trigonometry is indicated by the color’s red (C selection), green (S selection), and blue (R selection). Abbreviation explanation: R-(number/yr) means the number of years elapsed since restoration, TTS means temperate typical steppe, WS means forest steppe, and NR means non-restoration.

The CSR ecological strategies of the research sites consisted of a total of four types: R/CR (NR, R-7yr), R/CSR (R-6yr, R-13yr), S/SR (WS and R-10yr), and CSR (TTS) (Figure 4). As the restoration time elapsed in the damaged site (R/CR), the ecological strategies shifted from the strategic type R/CR to R/CSR and then to CSR at the sites where shrubs were introduced (R-7yr, R-6yr, R-13yr) similarly to the temperate typical steppe (TSS). On the other hand, at the site where trees and shrubs were introduced (R-10yr), the strategic types S/SR → S/SR (WS) appeared quite similarly to the woodland steppe (WS). Therefore, in the restoration of the damaged sites, succession progressed into two different types of ecological strategies according to the life-forms (shrub, tree) of the trees introduced in the early stage (Figure 4).

**Figure 5:**
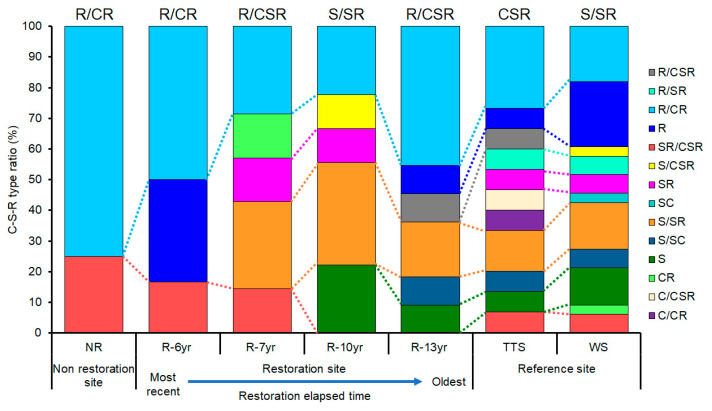
Ratio of types of C-S-R ecological strategies occupied from research site in Hulunbuir among a total of 19 CSR strategy classes proposed by Grime (2001). The black letters above the bar graph indicate the study site’s community CSR ecological strategy. Abbreviation explanation: R-(number/yr) means the number of years elapsed since restoration, TTS means temperate typical steppe, WS means woodland steppe, and NR means non-restoration.

Among the total of 14 types of C-S-R ecological strategies of plants by study site, the S-selective type and R-selective type were in all study sites, whereas the C-selective type appeared only at the TTS (Figure 5). The numbers of types of ecology strategies of plants that appeared by research site and the types that occupied the most were two and R/CR (75.0%) at NR, three and R/CR (50%) at R-6yr, five and R/CR and S/SR (28.6% each) at R-7yr, five and S/SR (33.3%) at R-10yr, S/SR (33.3%), and six and R/CR (45.4%) at R-13yr (Figure 5). In addition, in TTS and WS, there were 11 types of CSR ecological strategies, and the most common types were R/CR (29.4%) and S (25.7%), respectively. In NR and R-6yr, two to three types appeared, which were significantly less compared to the restored sites and reference ecosystems, and the longer the elapsed restoration time and in the reference ecosystem, the greater the number of types of CSR ecological strategies of the species that appeared (Figure 5).

**Figure 6:**
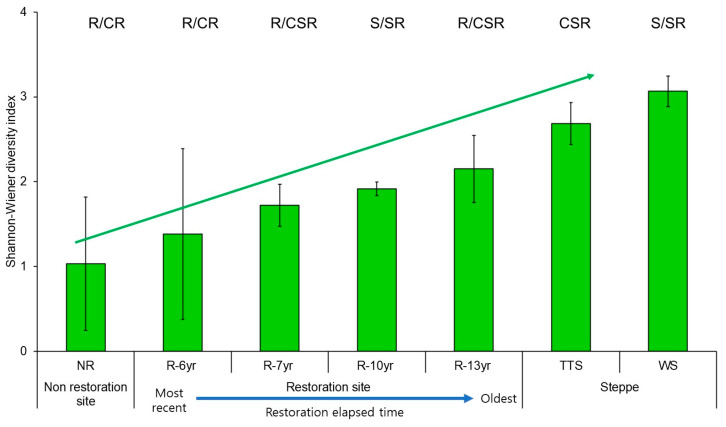
Comparison of Shannon–Wiener diversity of community CSR ecological strategy by research site in Hulunbuir. Error bars represent the standard deviation. The bars indicate the Shannon–Wiener diversity of the composition ratio of community CSR per study site. The blue dotted line above the bars of the unrestored and restored areas means the regression line (R^2^ = 98.5%). Abbreviation explanation: R-(number/yr) means the number of years elapsed since restoration, TTS means temperate typical steppe, WS means woodland steppe, and NR means non-restoration.

As vegetation was introduced to the damaged sites, the CSR eco-functional diversity of the communities increased in proportion to the elapsed time of restoration (Figure 6). The CSR eco-functional diversity was up to 2.1 times higher at the restored sites than at the unrestored sites and was 1.5 times higher at the restored site where the longest restoration time elapsed than at the site that was restored the most recently (Figure 6). In addition, the CSR eco-functional diversity was 0.8 times and 0.7 times lower at the site where the longest restoration time elapsed than at the reference ecosystems (TTS, WS), respectively (Figure 6).

### 3.3. Changes in Eco-Functional Diversity

**Figure 7:**
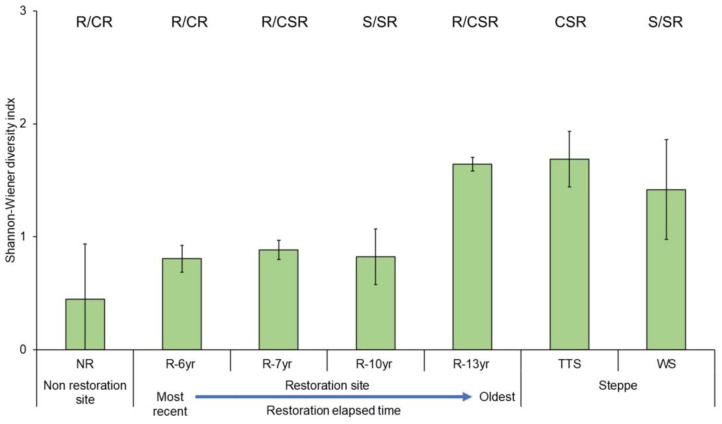
Comparison of Shannon–Wiener diversity of plant species ecological strategy by research site in Hulunbuir. Error bars represent the standard deviation. The bars indicate the Shannon–Wiener diversity of the composition ratio of community CSR per study site. The blue dotted line above the bars of the unrestored and restored area means the regression line (R^2^ = 75.5%). Abbreviation explanation: R-(number/yr) means the number of years elapsed since restoration, TTS means temperate typical steppe, WS means woodland steppe, and NR means non-restoration.

After the restoration of vegetation in the damaged sites, the eco-functional and taxonomic diversity increased over time (Figure 7 and Figure S2). The eco-functional diversity (H′) was the lowest at the unrestored site (0.45) and the highest at R-13yr (1.64), which was restored first, and the value reached 97% of the value at the steppe (TTS, 1.69) in the reference site. Also, the ecological diversity of the woody steppe (WS, 1.42) in the reference site was lower than that of the temperate typical steppe (TTS, 1.69).

In NR, the total number of species that appeared was 11 taxa, and the annual average number of species was the smallest, since it did not reach 10. On the other hand, in the reference ecosystems (TTS and WS), a total of 65 taxa appeared and the number of plant species was the largest among the research sites with an annual average at least 40 (Figure S2). In addition, among the average numbers of species at individual restored sites, those at the 13-year-old restored site (R-13yr) and the 10-year-old restored site (R-10yr), the oldest restored sites, were at least two times higher than those at the 7-year-oldrestored site (R-7yr) and the 6-year-oldrestored site (R-6yr), which were restored recently (Figure S2).

## 4. Discussion

### 4.1. Types of Plant CSR Ecological Strategies

A total of 14 types of ecological strategies were identified from the 56 taxa that appeared in the research sites, and CSR ecological strategies that are between the S-selective and the R-selective types accounted for 93% of the entire ecological strategies, and only the remaining three types (CR, C/CR, C/CSR) were the C-selective type (7.1%) (Figure 3). Among the primary, secondary, and tertiary types of plant CSR ecological strategies at all seven research sites, species with tertiary (R/CR, R/SR, S/SR) and primary (S and R) types were predominantly distributed in order of precedence (Figure 3 and Figure 5). Meanwhile, three types (CR, C/CR, C/CSR) were the C-selective type (7.1%), and species with the C-selective type were *Belamcanda chinensis*, *Populus canadensis*, *Patrinia rupestris*, and *Saussurea japonica* and appeared only at R-7yr, TTS, and WS where *Populus canadensis* was planted (Figure 3).

The climate of Hulunbuir is dry in winter, and 70% of the precipitation falls from June to August so that Hulunbuir is somewhat humid only during this time, but the climate is very dry since the annual precipitation is 274 mm, and at the same time, temperature differences and interannual fluctuations in precipitation are extremely large (Figure 1a). In addition, nutrients are poor since Hulunbuir is a sand dune and sand movements occurred frequently, and livestock were active and grazed in the whole area except for restored sites and reference ecosystems. Usually, the S-selective type is a stress-tolerant species, and it survives in habitats subject to resource stress due to severe resource deficiency [25,43,44,45]. On the other hand, the R-selective type opportunistically and rapidly settles down in habitats where resources are very abundant and external environmental factors change frequently, and these are frequently disturbed [25,43,44,45]. Thus, many species between S-selective and R-selective types appeared at the research sites, and it is attributable to the fact that environmental stress and disturbances are frequent there [27].

Usually, deep-rooted plants grow in arid areas such as deserts. Since the water demand of plants is higher in more arid environments, they must continue to take root in the water table to absorb water [25]. The reason why *Ulmus pumila*, which is a deep-rooted plant, was found to be extremely rarely in this study is that since this plant is an S/CSR type but has a strategy for environments where C, S, and R coexist, it is a deep-rooted plant that likes soils with appropriate wettability, although it is tolerant to environmental stress, and thus its ability to survive in the environments in the research sites decreased over time.

Among the restored species in the research sites, S-selective types are *P. sylvestris* var. *mongolica* (S) and *C. microphylla* (SR) (Figure 3). These two species were well established in the early stage and observed every year during the study period. S-selective type plants have a low specific leaf area and high dry weight content because they invest energy in leaves or roots. Since legumes such as *C. microphylla* should provide photosynthetic products to bacteria to maintain a symbiotic relationship with nitrogen-fixing bacteria, they should distribute some of their photosynthetic products to other functions. Therefore, they have a characteristic that their growth is generally slow [41,46,47]. It is thought that since they have the S ecological strategy, they developed tissues avoided by herbivores such as scale leaves in the case of *P. sylvestris* var. *mongolica* and thorns in the case of *C. microphylla* in the research sites to adapt to various stress environments and increase the survival rate [37]. In addition, the scale leaves of *P. sylvestris* var. *mongolica* are a strategy to increase the survival rate in dry conditions thanks to their ability to reduce transpiration to the minimum, and this is judged to be the reason why it grows well in the research sites [48,49]. On the other hand, *C. microphylla* is considered to have grown well in the research sites because it has a strategy to adapt well to habitats with low productivity or barren environments through symbiosis with nitrogen-fixing bacteria [50]. Therefore, it can be seen that these two are suitable as tree species in the early stage of restoration.

The CSR ecological strategy of *C. fruticosum*, a restored species, was the R-selective type, which is an R/CR type (Figure 3), and it was observed in all research sites including NR, restoration sites and reference ecosystems. The reason why *C. fruticosum* could settle down well in the research sites is thought to be the fact that it has the ability to settle opportunistically and quickly in harsh, dry climate environments where sand movements are frequent, soil resources change frequently, and disturbances (livestock and humans) are frequent given the strategic characteristics of R-selective and CR (competitive ruderal species) types [45]. Also, *C. fruticosum* seems to be highly preferred by animals since its flowering time is early, its flowering period is long, and its leaves are large because many resources are distributed to them. Since the forgoing condition is connected to breeding [25], settlement is expected to be good in all research sites. *C. fruticosum* has been observed every year at unrestored sites, restored sites, and even in reference ecosystems because it had a competitive advantage against disturbances and for resources, since its ecological strategy ratio was C:S:R = 19:0:81%. It has been predominantly observed annually. Therefore, *C. fruticosum* is thought to be appropriate as a species to be restored in the research sites because it endures well since it has competitive strategies even though it is an R-selective type and is tolerant in competition with later species.

A wasteland is an environment where most organisms can hardly live due to sand movement, the lack of soil nutrients, etc. Nevertheless, pioneer plants that first enter desert areas and live by overcoming unfavorable conditions for growth and development such as the lack of nutrients, dryness or dampness, and light [51] and nursing plants that help and protect the resettlement of other plants [52] make the environment favorable for other species to live [25]. In the restored sites in this study, both pioneer plants (*Leymus secalinus* and *Corispermum hyssopifolium* (R/CR)) and nursing plants (*C. microphylla* (S/SR) and *C. fruticosum* (R/CR)) used as restoration planting species were distributed, and *C. hyssopifolium* and *C. microphylla* appeared even in NR where there was little vegetation (Figure 3). Since there were pioneer and nursing species, it seems that they affected the development of eco-functional diversity according to the restoration period. Considering the aspects of conservation and prevention of desertification of the Hulunbuir grassland ecosystem, which has been undergoing desertification as the grassland area has recently decreased, the preservation and utilization of pioneer species and nursing species with ecological strategies that can lead to increases in ecological functional diversity by helping the introduction and settlement of plants seem to be very important.

*Populus canadensis* with the CR-type ecological strategy was planted in R-7yr, but it has not rooted well, and withering individuals even appeared (author’s observation). The C-selective species live in resource-rich places, distribute most of their photosynthetic products to growth, and have broad leaves, large amounts of biomass, and highly competitive strategies for resources [43,44,45]. Therefore, the reason why they did not grow well and did not survive to the end is that their vitality gradually decreased due to their low ecological status in the research sites, where there were many environmental stresses and disturbances. Therefore, *P. canadensis* with the C-selective type of the CR type is judged to be suitable as a mid- and late-stage restoration tree species rather than an early one.

In addition, genera such as *Allium*, *Artemisia*, *Corispermum*, and *Potentilla* appeared more than twice in the research sites, but their strategies were different even if they were in the same genus (Figure 3). Five genera that were the same as those in this study appeared in semi-natural xeric calcareous grasslands in Italy, but their ecological strategies happened differently, such as the genus *Sanguisorba,* which was R/SR in this study but was SC in Italy, and the genus *Festuca,* which was S/SR in this study and appeared to be S, which is the same primary type, in the literature [53]. Given the foregoing, it is judged that the plants appeared identically or differently even if they were in the same genus because they can have very diverse ecological strategies by responding sensitively to environmental changes [54].

The reason why plant CSR ecological strategies are mainly stress-tolerant (S) and ruderal species (R) rather than competitive (C) ecological strategies is predicted to be the fact that since the environment where the study subject plants live is an area where soil moisture is especially poor and various disturbances such as those caused by herbivores or humans frequently occur, strategies with tolerance to moisture and drying are mainly distributed in the environment. In deserts, forelands, and alpine meadows where stress environments occur due to frequent disturbances, limited resources, and in particular, the occurrence of seasonal precipitation, species with S-selective and R-selective types, which are two extremes, were mainly distributed similarly to the types of plant CSR ecology strategies that appeared in this study [55,56,57] consistently with the results of this study. Therefore, it is judged that the CSR ecological strategies can be used as an index representing the situation of the region through a comprehensive analysis of the functional characteristics of plant species to evaluate tree species suitable for restoration.

### 4.2. Changes in Community CSR Ecological Strategies and Eco-Functional Diversity Following the Progress of Restoration

Globally, a total of four types of CSR ecological strategies of communities (CS/CSR, SR/CSR, CSR, R/CSR) appeared in the grassland ecosystem, and the type of ecological strategy of the TTS in Hulunbuir was CSR (C:S:R = 27:26:47) (Figure 4). The grasslands that coincided with the research sites in this study were flooded grasslands and savannas, but the ratios of diversity did not completely coincide because the constituent species of these places were mainly C-selective and S-selective types, and these types were abundant [27]. On the other hand, the grassland where the ratios of diversity of constituent species between R-selective types and S-selective types was like those in this study was the montane grassland (R/CSR), but the types of ecological strategies were different from those in the research sites in this study [27]. Also, the ecological strategies of temperate grasslands (SR/CSR) with the same grassland type were different from those of the Hulunbuir steppe [27]. Grassland ecosystems have various types of community CSR ecological strategies, and even in the case of the same biomes, the ecological strategies were different from each other. The reasons for this are judged to be the fact that grassland ecosystems are a type of vegetation that is sensitive to environmental changes [17], and among the environmental factors that divide grassland types, precipitation in particular is the cause [58].

The life history strategies of plants are greatly affected by environmental factors such as topographic changes, soil environment development, and increased light intensity, and plants living in one space have similar life history strategies [12]. The types of CSR ecological strategies of the grassland ecosystem in the research sites were largely divided into temperate typical steppe (CSR) and woodland steppe (S/SR) according to the species restored in the damaged grasslands (Figure 4), and the results show that two types of grassland vegetation exist in the field (Figure 4). The movements of the ecological strategies following the progress of succession restored in the damaged land were R/CR → R/CSR in order of precedence in the restored site where the initially introduced species was shrubs, similarly to those of the temperate typical steppe (CSR). On the other hand, the type of ecological strategies of the places where high trees were introduced was S/SR closely to the woodland steppe (Figure 4). The introduction of vegetation increased the number of plant CSR ecological strategies and ecological functional diversity in proportion to the elapsed restoration time (Figure 5 and Figure 6). After introducing vegetation into damaged sites and they shifted from unrestored sites to restored sites and approached the reference ecosystem, the functional diversity of the CSR ecological strategy increased up to three times (Figure 6). Thus, species constituting functional types of plant communities develop while sharing similar or identical ecological strategy attributes [26,45]. This trend can also be seen on the triangular diagram. The types of community CSR ecological strategies of the TTS and the WS were CSR and SS/SR, respectively, and the distance between them was 0.4619, indicating that they were completely different. It is judged that the community CSR ecological strategies were divided into two due to the selection of the species to be restored and the mutual relationship between the constituent species and environmental factors according to the lapse of restoration time.

The research sites that were grouped into grassland ecosystems are the ones where R-selective shrubs were planted (R-6yr, R-7yr, R-13yr) and CSR-type TTS. Here, the CSR ecological strategies moved from NR to approach TTS (Figure 4). The results coincided with the trend for the distance to the TTS in the arrangement of community ecological strategies to be larger in the cases of R-7yr (0.1247) and R-13yr (0.1440) than in the case of NR (0.3107) and R-6yr (0.3407) (Figure 4). The results coincided with the result where the ecological and functional diversity of the unrestored land increased by two times at the maximum as the restoration time elapsed after the introduction of vegetation (Figure 6).

The types of CSR ecological strategies of NR and R-6yr were R/CR types that were intensively distributed toward the R-selective type, and the distance between the two regions was 0.0459 (Figure 4). Although the CSR ecological strategy of most of the constituent species of these two vegetations was R-selective of the annual species, similar community CSR ecological strategies appeared between the two regions because *C. fruticosum*, a slow-growing perennial plant, was dominant. It is also judged that the plant species diversity is low and plant community development is slow in this area because it is a temporary habitat where disturbances occur, and it has environmental conditions that make it difficult for plants to live. The distance between R-7yr and R-13yr, which are R/CSR types, was 0.0690, and the reason why similar types appeared (Figure 4) is that as *C. microphylla* or *C. fruticosum* became dominant, this area came to have similar ecological strategies. In addition, the distance between R-6yr and R-7yr was shown to be 0.2173, indicating that large differences in community CSR ecological strategies appeared from 6 years after restoration (Figure 4). The reason is considered to be that because the R-13yr and R-7yr restored sites were dispersed to the R and S strategies, unlike the NR and R-6yr, which were arranged near the R strategy, and the distance between the communities was shorter in the case of NR and R than in the case of NR and R-6yr, this site was close to TTS, which is the reference site (Figure 4).

In the case of the research sites grouped into the grassland ecosystem, *C. microphylla* and *C. fruticosum* were mainly planted as initial restoration tree species. These two species were mainly dominant in the research sites and were observed every year during the study period. The reason why they grew well is the strategy to increase adaptability and survival rate through symbiosis with nitrogen-fixing bacteria. Since species with these strategies were used as restoration species in devastated areas, they could continue to help the introduction and settlement of various plant species or species with similar strategies. Therefore, it is thought that these research sites are moving toward temperate typical steppes with the most stable ecological strategy because the richness of constituent species developed evenly as time passed after restoration.

The woodland grasslands were divided into restored sites where trees were planted (S/SR) and woodland steppes (S/SR), and the distance between them was 0.0783 (Figure 4). The reason for this is that they were grouped into similar types because they belong to the same dominant species (*P. sylvestris* var. *mongolica*) group. Therefore, the restoration site of R-10yr is advancing toward the restoration goal since it is approaching WS. However, in R-10yr, the abundance of *C. microphylla* Lam was somewhat high, and since the coverage in the lower layer and the supply of excessive resources could rather reduce species richness [12], it seems that the foregoing should be periodically monitored and observed along with the growth status of the constituent species. In addition, since S-type plants grow slowly and their leaves decompose slowly even in favorable conditions due to various characteristics, they can affect the circulation of substances, which is important in the ecosystem [26]. Therefore, it is judged that the strategy should be improved to make dispersed combinations of the research sites groups into woodland steppe and other types so that they are not concentrated only on the S type.

The development of increased eco-functional diversity with the introduction of vegetation into the damaged sites in this study is expected to have the largest effects on the development of the eco-functional groups of plants growing in the same microenvironment according to the elapsed time of restoration, changes from floating sand dunes to fixed sand dunes, improvement of soil holding capacity, increases in soil nutrients, etc. In fact, the change in the topography of Horqin sandy land in Hulunbuir, which was a grassland in the past, led to changes in dominant species and decreases in plant species diversity and eventually, *C. microphylla* and *Setaria viridis*, which were tree species at the early stage when the decline in vegetation did not progress very much, were replaced by *C. microphylla* and *S. viridi.* This decline in vegetation progressed severely so the land soon became wastelands [20]. When considered from the perspective of recovery of the vegetation in the restored sites, *Artemisia halodendron* (SR), an annual herb, appeared in the R-7yr and R-10yr restoration sites, which are semi-fixed dunes, *C. hyssopifolium* (R/CR), an annual herb, was found in R-6yr, R-7yr and R-10yr, *C. microphylla* (S/SR), a perennial shrub, was found in R-7yr, R-10yr and R-13yr, and *A. cristatum* (S/SC), a perennial herb, was found only in R-13yr (Figure S3). Also, sand dunes changed into fixed dunes in R-13yr, so the topography and soil were restored. These results indicate that changes in plant life cycle strategies are related to environmental changes, since changes in plant species composition according to restoration time in the research sites can be seen as the opposite of Horqin’s vegetation reduction.

### 4.3. Changes in Plant Species Diversity According to the Progress of Restoration

Plant species diversity was the lowest in NR and the highest in TTS, and among restored sites, it was the highest in R-13yr with the longest restoration time, where the level of plant species diversity was like that of grasslands (Figure 7). With an average of 10 taxa or more, the restoration sites had larger numbers of species than the unrestored sites, and the plant diversity in the areas where more than 10 years had passed since restoration increased more than twice compared to areas where 6 to 7 years had passed. The phenomenon of increases in the appearance of Hulunbuir native species seems to clearly show the results of restoration [59]. Identically to this study, the introduction of vegetation into damaged lands increased plant species diversity in restored areas compared to wasteland degraded areas, thereby proving the effect of vegetation restoration [60,61,62]. In addition, plant species diversity in other Hulunbuir dry and semi-arid grasslands and woodland steppes increased with wetting and was seven times higher in wet mesic grassland than in damaged lands [63]. Similarly, in this study, when drylands were considered as NR and semi-arid grasslands and forest grasslands were considered as restoration sites and reference ecosystems, respectively, plant species diversity was shown to be three times higher at the oldest restored site than NR and five times higher in the reference ecosystem (TTS, WS) than NR (Figure S1). In view of this, this study demonstrated the restoration effect of the introduction of vegetation, as it increased plant species diversity.

Also, stock farming plays an important role in the rural economy of Inner Mongolia, and natives are particularly interested in plants that can be used as food sources for animals that are directly related to their lives [5]. In Hulunbuir, most of the poaceae and legumes, including *Leymus,* which are rich in nutrients in underground stems, are used as food sources for animals [64]. In the research sites, plants that can be used as food sources were shown to be *Leymus chinensis*, *Agropyron cristatum*, *Calamagrostis epigeios*, *Cleistogenes squarrosa*, *C. fruticosum*, and *Stipa sareptana* var. *krylovii* [40]. Among the plants used for vegetation restoration, *C. microphylla* and *C. fruticosum* were detected in the feces of livestock living in the research sites [52], indicating that they are eaten by animals. Given the foregoing, the introduction of native tree species and useful resource vegetation to damaged grasslands increases plant species diversity so that many species can be used as food sources. Therefore, the damaged grasslands are becoming an environment where wild animals can live, and it is predicted that as plants are gradually diversified, the diversity of the fauna will increase. In addition, the increase in food sources for livestock through vegetation restoration is judged to be of great significance because it not only has a great impact on the restoration effect by continuously inducing active participation in restoration and enhancing restoration awareness but is also directly related to the lives of residents.

## 5. Conclusions

This study is the first attempt to identify the movements of the CSR ecological strategies and the increase in eco-functional diversity in Hulunbuir grasslands in Asia with the analysis of CSR ecological strategies. The introduction of trees in the damaged grasslands increased the diversity of eco-functional groups and led to the transition to temperate typical grasslands when the life-form of the initially introduced plants was shrubs and to forest grasslands in the reference site when the life-forms of the initially introduced plants were trees and shrubs. Thus, the development of the diversity of CSR ecological strategies in proportion to the passage of time in the restored sites means that the restoration of vegetation in the damaged sites has been successfully achieved, and this succession series may vary depending on the life-form of the introduced woody plants. Therefore, in the restoration of damaged lands in the steppe, it is thought that plants should be selected and introduced by considering the final goal of restoration (grass steppe, woodland steppe) first.

For the successful ecological restoration of damaged grasslands, above all, the participation of residents in Hulunbuir and the promotion of positive awareness of the restoration project played a great role. This is because the food source for livestock desired by residents has increased through the restoration project.

When considering these points, it is thought desirable to set the temperate typical steppe (TSS), where plants are used as a food source for livestock, as the final goal of restoration rather than the woodland steppe (WS). To this end, planting shrubs such as *C. microphylla* and *C. fruticosum* as initial restoration species introduced will lead to herbaceous steppes more quickly. In addition, to prevent damage to introduced vegetation caused by sand movement here, the installation of fences using reeds or straw is an important consideration for successful ecological restoration.

Also, it is meaningful in that it predicted the direction of C-S-R of the restored area by identifying and comparing the strategic attributes of each community (degraded steppe, restored area, and reference steppe) based on the distance difference between C-S-R ecological strategies. In the future, it is expected to be used as comparative data when investigating the C-S-R ecological strategies of plant species and communities in restored sites or as basic data for selecting tree species for planting in damaged areas.

## Figures and Tables

**Table 1:** Characteristics of the study site in Hulunbuir.

Characteristic of Site	Site * Name	Topology Type	Elevation (m)	Planted Method	Introduced or Dominant Plant (Life-Form)	Abundance State (Coverage, %)
Non-restoration	NR	Shifted sandy dune	601	-	*Corethrodendron fruticosum* B.H.Choi & H.Ohashi (Shrub)	3.1
Restoration	R-6yr	Sand dune	590	Shrub planting + Seed spray	*C. fruticosum* (Shrub)	42.7
	R-7yr		590	Tree + Shrub planting	*Populus canadensis* Moench (Tree) *C. fruticosum* (Shrub)	43.7
	R-10yr		575		*Pinus sylvestris* var. *mongolica* Litv. (Tree) *Caragana microphylla* Lam. (Shrub)	43.9
	R-13yr		581	Shrub planting + Seed spray	*C. fruticosum* (Shrub)	74.7
Reference	TTS	Fixed sandy dune	693	-	*Galium verum* var. *asiaticum* (Nakai) T. Yamaz. (Herb)	77.1
	WS		729	-	*P. sylvestris* var. *mongolica* (Tree)	51.3

* Abbreviation explanation: R-(number/yr) means the number of years elapsed since restoration, TTS means temperate typical steppe, WS means woodland steppe, and NR means non-restoration.

## Data Availability

The data presented in this study are available in the Supplementary Material.

## Supplementary Materials

The following supporting information can be downloaded at https://www.mdpi.com/article/10.3390/biology12121479/s1, Figure S1: Scientific names and CSR ecological strategies of plants that appeared in Hulunbuir research site. The photograph located in the center of the triangle graph shows a plant, and the C-S-R strategy location is indicated by a circle above the line; Figure S2: Comparison of plant biodiversity by research site in Hulunbuir for 5 years (2014–2018). Black dots represent the average number of plant species detected and error bars represent the standard deviation. Blue bars mean the total number of species present and alphabet in blue bar mean differences in mean number of species per study site. NR: site of non-restoration, R-(number/yr): years of time elapsed since restoration. Steppe includes both temperate typical steppe (TTS) and woodland steppe (WS); Figure S3: The vegetation decline of desertification areas of Horqin [20] and process of ecosystem restoration according to the vegetation restoration of Hulunbeier in Inner Mongolia.

## References

[1] Park B.K. (1985). Korea’s Grassland Research.

[2] White R., Murray S., Rohweder M. (2000). Pilot Analysis of Global Ecosystems: Grassland Ecosystems.

[3] Shantz H.L. (1954). The place of grasslands in the Earth’s cover. Ecology.

[4] Eyre S.R. (1968). Vegetation and Soils: A World Picture.

[5] Angerer J., Han G., Fujisaki I., Havstad K. (2008). Climate change and ecosystems of Asia with emphasis on Inner Mongolia and Mongolia. Rangelands.

[6] Wrage N., Strodthoff J., Cuchillo H.M., Isselstein J., Kayser M. (2011). Phytodiversity of temperate permanent grasslands: Ecosystem services for agriculture and livestock management for diversity conservation. Biodivers. Conserv..

[7] Li M., Wang X., Chen J. (2022). Assessment of grassland ecosystem services and analysis on its driving factors: A case study in Hulunbuir Grassland. Front. Ecol. Evol..

[8] Blair J., Nippert J., Briggs J. (2014). Grassland ecology 14. Ecol. Environ..

[9] Chen Y., Li Y., Zhao X., Awada T., Shang W., Han J. (2012). Effects of grazing exclusion on soil properties and on ecosystem carbon and nitrogen storage in a sandy rangeland of Inner Mongolia, Northern China. Environ. Manag..

[10] Chen L., Gao J., Ji Y., Bai Z., Shi M., Liu H. (2014). Effects of particulate matter of various sizes derived from suburban farmland, woodland and grassland on air quality of the central district in Tianjin, China. Aerosol Air Qual. Res..

[11] Montanarella L., Scholes R., Brainich A. (2018). The Assessment Report on Land Degradation and Restoration.

[12] Li J., Zheng Z., Xie H., Zhao N., Gao Y. (2017). Increased soil nutrition and decreased light intensity drive species loss after eight years grassland enclosures. Sci. Rep..

[13] Deák B., Rádai Z., Lukács K., Kelemen A., Kiss R., Bátori Z., Péter János K., Valkó O. (2020). Fragmented dry grasslands preserve unique components of plant species and phylogenetic diversity in agricultural landscapes. Biodivers. Conserv..

[14] Oikawa T., Ito A., Matsuno T., Kida H. (2001). Modeling carbon dynamics of terrestrial ecosystem in monsoon Asia. Present and Future of Modeling Global Environmental Change: Towards Integrated Modeling.

[15] Akiyama T., Kawamura K. (2007). Grassland degradation in China: Methods of monitoring, management, and restoration. Grassl. Sci..

[16] Zhang Z., Huisingh D. (2018). Combating desertification in China: Monitoring, control, management and revegetation. J. Clean. Prod..

[17] Yang B., Gong J.R., Zhang Z.H., Wang B., Zhu C.C., Shi J., Liu M., Liu Y., Li X. (2019). Stabilization of carbon sequestration in a Chinese desert steppe benefits from increased temperatures and from precipitation outside the growing season. Sci. Total Environ..

[18] Li W.J., Ali S.H., Zhang Q. (2007). Property rights and grassland degradation: A study of the Xilingol Pasture, Inner Mongolia, China. J. Environ. Manag..

[19] Kang L., Han X., Zhang Z., Sun O.J. (2007). Grassland ecosystems in China: Review of current knowledge and research advancement. Philos. Trans. R. Soc. B Biol. Sci..

[20] Squires V.R., Lu X., Lu Q., Wang T., Yang Y. (2009). Chapter 7: Case study1 Hulunbeier grassland, Inner Mongolia. Rangeland Degradation and Recovery in China’s Pastoral Lands.

[21] Batunacun, Hu Y., Biligejifu, Liu J., Zhen L. (2015). Spatial distribution and variety of grass species on the Ulan bator—Xilinhot transect of Mongolian plateau. J. Nat. Resour..

[22] McIntosh R.P. (1986). The Background of Ecology: Concept and Theory.

[23] Bai X., Zhao W., Wang J., Ferreira C.S.S. (2022). Reducing plant community variability and improving resilience for sustainable restoration of temperate grassland. Environ. Res..

[24] Fry E.L., Savage J., Hall A.L., Oakley S., Pritchard W.J., Ostle N.J., Pywell R.F., Bullock J.M., Bardgett R.D. (2018). Soil multifunctionality and drought resistance are determined by plant structural traits in restoring grassland. Ecology.

[25] Barbour R.K.K., Burk P., Pitts J., Gilliam F., Schwartz M. (2015). Terrestrial Plant Ecology.

[26] Hunt R., Hodgson J.G., Thompson K., Bungener P., Dunnett N.P., Askew A.P. (2004). A new practical tool for deriving a functional signature for herbaceous vegetation. Appl. Veg. Sci..

[27] Pierce S., Negreiros D., Cerabolini B.E.L., Kattge J., Díaz S., Kleyer M., Shipley B., Wright S.J., Soudzilovskaia N.A., Onipchenko V.G. (2017). A global method for calculating plant CSR ecological strategies applied across biomes world-wide. Funct. Ecol..

[28] Grime J.P., Pierce S. (2012). The Evolutionary Strategies that Shape Ecosystems.

[29] Yu J., Hou G., Zhou T., Shi P., Zong N., Sun J. (2022). Variation of plant CSR strategies across a precipitation gradient in the alpine grasslands on the northern Tibet Plateau. Sci. Total Environ..

[30] Zhu Y., Shan D., Wang B., Shi Z., Yang X., Liu Y. (2019). Floristic features and vegetation classification of the Hulun Buir steppe in North China: Geography and climate-driven steppe diversification. Glob. Ecol. Conserv..

[31] Walter H., Harnickell E., Mueller-Dombois D. (1975). Climate Diagram Maps of the Individual Countries and the Ecological Climatic Regions of the Earth: Supplement to the Vegetation Monographs.

[32] (2023). China Meteorological Data Service Centre. http://data.cma.cn/.

[33] Whittaker R.H. (1962). Classification of natural communities. Bot. Rev..

[34] Whittaker R.H. (1970). Communities and Ecosystems.

[35] Kottek M., Grieser J., Beck C., Rudolf B., Rubel F. (2006). World Map of the Köppen-Geiger climate classification updated. Meteorol. Z..

[36] Park K.H., Qu Z.Q., Wan Q.Q., Ding G.D., Wu B. (2013). Effects of enclosures on vegetation recovery and succession in Hulunbeier steppe, China. For. Sci. Technol..

[37] Hodgson J.G., Wilson P.J., Hunt R., Grime J.P., Thompson K. (1999). Allocation C-S-R plant functional types: A soft approach to a hard problem. Oikos.

[38] Gu A., Wang Z. (2009). Atlas of Rangeland Plants in Northern China.

[39] Gu A., Wang Z. (2011). Atlas of Rangeland Plants in Northern China (Supplement).

[40] (2023). Flora of China. http://www.efloras.org/.

[41] Pierce S., Brusa G., Vagge I., Cerabolini B.E.L. (2013). Allocations CSR plant functional types: The use of leaf economics and size traits to classify woody and herbaceous vascular plants. Funct. Ecol..

[42] Shannon C.E. (1948). A mathematical theory of communication. Bell Syst. Tech. J..

[43] Grime J.P. (1977). Evidence for the existence of three primary strategies in plants and its relevance to ecological and evolutionary theory. Am. Nat..

[44] Grime J.P., Bradshaw A.D., Goode D.A., Thorpe E. (1986). Manipulation of plant species and communities. Ecology and Design in Landscape.

[45] Grime J.P. (2001). Plant Strategies and Vegetation Processes and Ecosystem Properties.

[46] Grime J.P., Hunt R. (1975). Relative growth-rate: Its range and adaptive significance in a local flora. J. Ecol..

[47] Tilman D. (1998). Plant Strategies and the Dynamics and Structure of Plant Communities.

[48] Liu Y., Bao G., Song H., Cai Q., Sun J. (2009). Precipitation reconstruction from Hailar pine (*Pinus sylvestris* var. *mongolica*) tree rings in the Hailar region, Inner Mongolia, China back to 1865 AD. Palaeogeogr. Palaeoclimatol. Palaeoecol..

[49] Song L., Zhu J., Zhang J., Zhang T., Wang K., Wang G., Liu J. (2019). Effect of drought and topographic position on depth of soil water extraction of *Pinus sylvestris* L. var. *mongolica* Litv. trees in a semiarid sandy region, Northeast China. Forests.

[50] Bessle H., Oelmann Y., Roscher C., Buchmann N., Scherer-Lorenzen M., Schulze E.D., Temperton V.M., Wilcke W., Engels C. (2012). Nitrogen uptake by grassland communities: Contribution of N_2_ fixation, facilitation, complementarity, and species dominance. Plant Soil.

[51] Tang L., Mao L., Shu J., Li C., Shen C., Zhou Z. (2020). Atlas of Quaternary Pollen and Spores in China.

[52] Kim J.H., Choi S.S., An I.J., Lee S.H., Lee E.J., You Y.H., Kim B.J., Han D.U., Park S.K., Joo S.B. (2021). Palatability and livestock preferences of restored plants in steppe restoration areas, Hulunbuir, Inner Mongolia, China. Proc. Natl. Inst. Ecol. Repub. Korea.

[53] Pierce S., Vagge I., Brusa G. (2014). The intimacy between sexual traits and Grime’s CSR strategies for orchids coexisting in semi-natural calcareous grassland at the Olive Lawn. Plant Ecol..

[54] Critchley C.N.R. (2000). Ecological assessment of plant communities by reference to species traits and habitat preferences. Biodivers. Conserv..

[55] Caccianiga M., Luzzaro A., Pierce S., Ceriani R.M., Cerabolini B. (2006). The functional basis of a primary succession resolved by CSR classification. Oikos.

[56] Pierce S., Ceriani R.M., De Andreis R., Luzzaro A., Cerabolini B. (2007). The leaf economics spectrum of *Poaceae* reflects variation in survival strategies. Plant Biosyst..

[57] Zhou T., Hou G., Sun J., Zong N., Shi P. (2021). Degradation shifts plant communities from S-to R-strategy in an alpine meadow, Tibetan Plateau. Sci. Total Environ..

[58] Shimoda S., Mo W., Oikawa T. (2005). The effects of characteristics of Asian monsoon climate on interannual CO_2_ exchange in a humid temperate C3/C4 co-occurring grassland. Sci. Online Lett. Atmos..

[59] Zhang W. (1998). Changes in species diversity and canopy cover in steppe vegetation in Inner Mongolia under protection from grazing. Biodivers. Conserv..

[60] Lv S.H., Feng C.S., Gao J.I., Lu X.S. (2008). Study on enclosing effects and biodiversity variation of desertification grassland in Hulunbeir Steppe. Acta Agrestia Sin..

[61] Quan Q., Nianpeng H., Zhen Z., Yunhai Z., Yang G. (2015). Nitrogen enrichment and grazing accelerate vegetation restoration in degraded grassland patches. Ecol. Eng..

[62] Yao M., Rui J., Li J., Wang J., Cao W., Li X. (2018). Soil bacterial community shifts driven by restoration time and steppe types in the degraded steppe of Inner Mongolia. Catena.

[63] Amartuvshin N., Kim J.B., Cho N.H., Seo B.S., Kang S.K. (2022). Local and regional steppe vegetation palatability at grazing hotspot areas in Mongolia. J. Ecol. Environ..

[64] Zhu M.J., Ren A.Z., Wen W., Gao Y.B. (2013). Diversity and taxonomy of endophytes from *Leymus chinensis* in the Inner Mongolia steppe of China. FEMS Microbiol. Lett..

