# Supplementary material for: Changes in Competitors, Stress Tolerators, and Ruderals (CSR) Ecological Strategies after the Introduction of Shrubs and Trees in Disturbed Semiarid Steppe Grasslands in Hulunbuir, Inner Mongolia
Source: Biology (Basel). 2023 Nov 30;12(12):1479. doi: 10.3390/biology12121479 (PMC10740407; doi:10.3390/biology12121479)
Supplement: Supplementary file 1 [file biology-12-01479-s001.zip › biology-2657407-supplementary.pdf]

Supplementary Materials

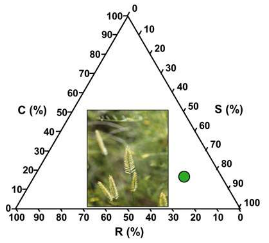

Agropyron cristatum **(L.) Gaertn.**
(S/SC)

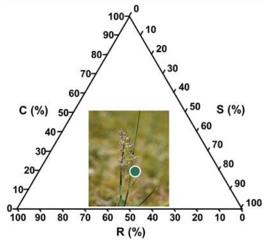

Agrostis alba **L.**
(SR/CSR)

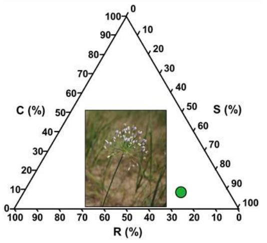

Allium mongolicum **Regel**
(S/SR)

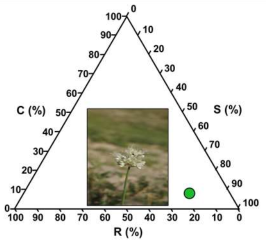

Allium senescens **L.**
(S/SR)

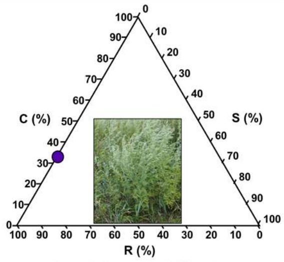

Artemisia capillaris **Thunb.**
(R/CR)

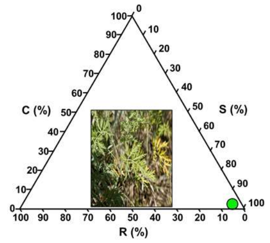

Artemisia caruifolia **Buch.–Ham. ex Roxb.**
(S)

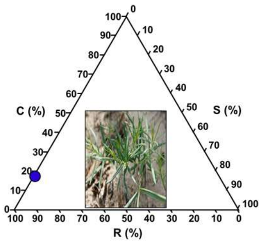

Artemisia desertorum **Spreng.**
(R/CR)

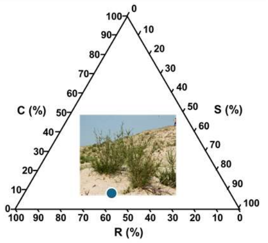

Artemisia halodendrom **Turcz. ex Bess.**
(SR)

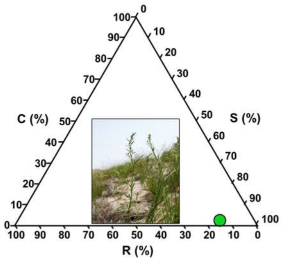

Artemisia scoparia **Waldst. & Kit.**
(S)

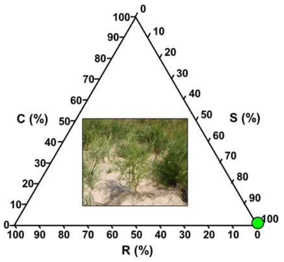

Asparagus dauricus **Fisch. ex Link**
(S)

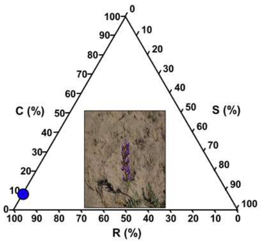

Astragalus laxmannii **Jacq.**
(R)

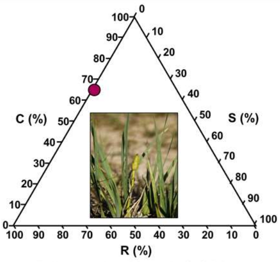

Belamcanda chinensis **(L.) DC.**
(C/CR)

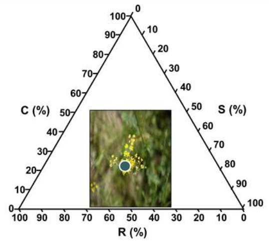

Bupleurum scorzonerifolium **Willd.**
(SR/CSR)

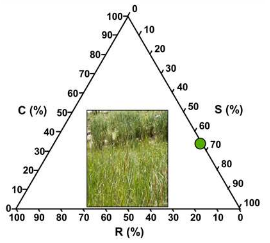

Calamagrostis epigeios **(L.) Roth**
(S/SC)

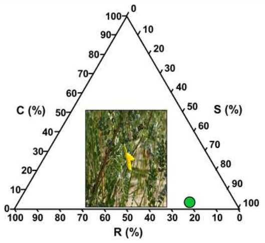

Caragana microphylla **Lam.**
(S/SR)

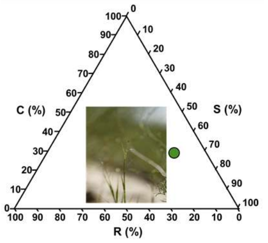

Carex korshinskyi **Kom.**
(S/CSR)

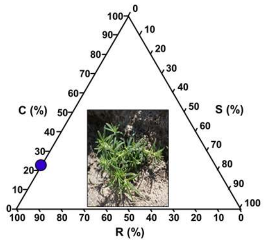

Chenopodium aristatum **L.**
(R/CR)

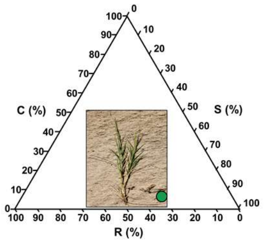

Cleistogenes squarrosa **(Trin.) Keng**
(S/SR)

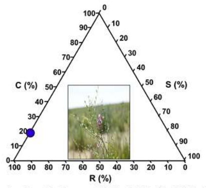

Corethrodendron fruticosum **(Pallas) B.H. Choi & H. Ohashi**
(R/CR)

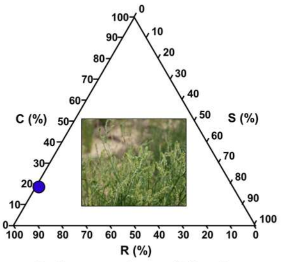

Corispermum hyssopifolium **L.**
(R/CR)

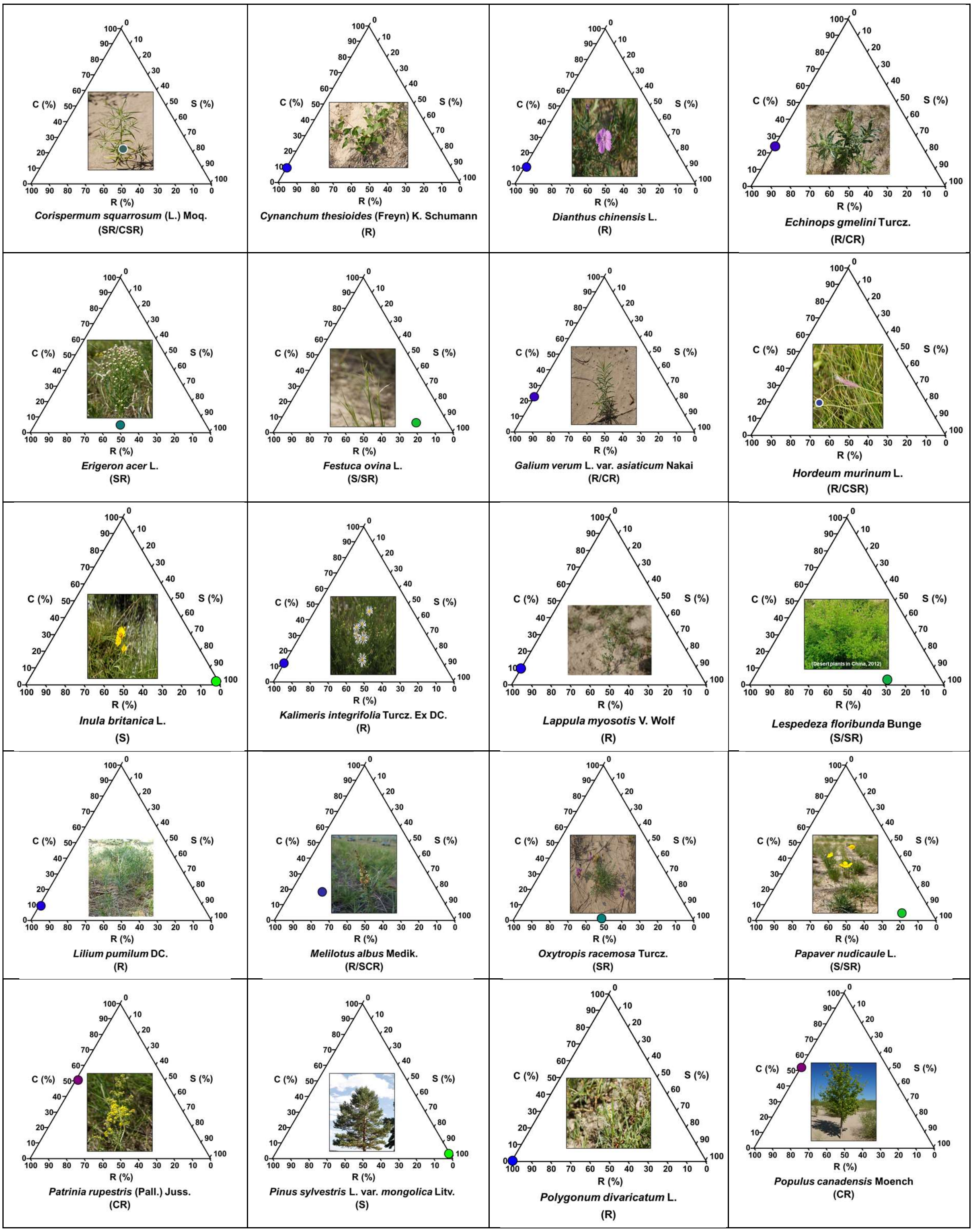
C (%)
S (%)
R (%)
Corispermum squarrosum (L.) Moq.
(SR/CSR)
Cynanchum thesioides (Freyn) K. Schumann
(R)
Dianthus chinensis L.
(R)
Echinops gmelini Turcz.
(R/CR)
Erigeron acer L.
(SR)
Festuca ovina L.
(S/SR)
Galium verum L. var. asiaticum Nakai
(R/CR)
Hordeum murinum L.
(R/CSR)
Inula britanica L.
(S)
Kalimeris integrifolia Turcz. Ex DC.
(R)
Lappula myosotis V. Wolf
(R)
(Desert plants in China, 2012)
Lespedeza floribunda Bunge
(S/SR)
Lilium pumilum DC.
(R)
Melilotus albus Medik.
(R/SCR)
Oxytropis racemosa Turcz.
(SR)
Papaver nudicaule L.
(S/SR)
Patrinia rupestris (Pall.) Juss.
(CR)
Pinus sylvestris L. var. mongolica Litv.
(S)
Polygonum divaricatum L.
(R)
Populus canadensis Moench
(CR)

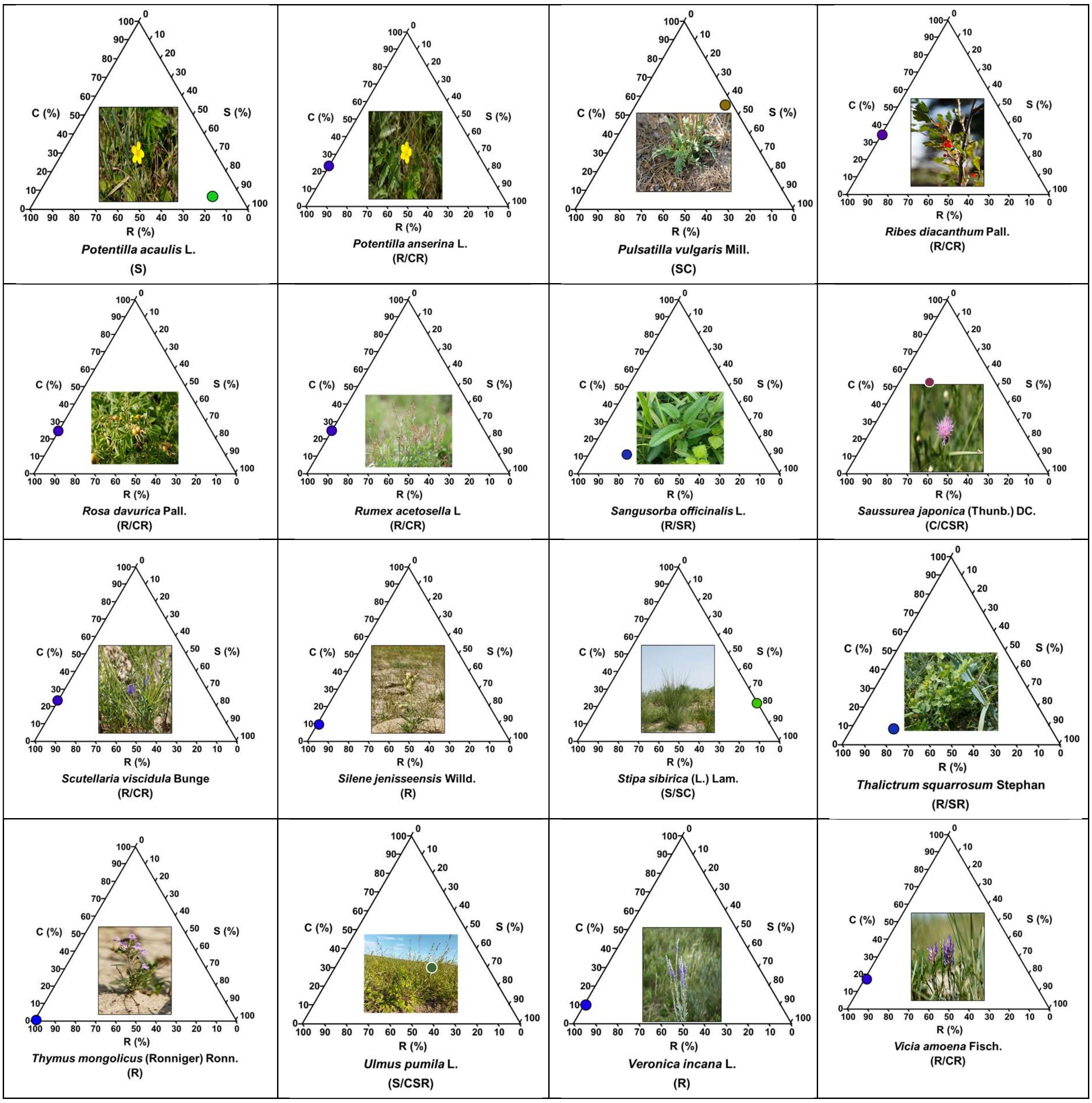

Figure S1. Scientific names and CSR ecological strategy types of plants that appeared in Hulunbuir research site. The photo located in the center of the triangle graph shows a photo of the plant, and the C-S-R strategy location is indicated by a circle above the line.

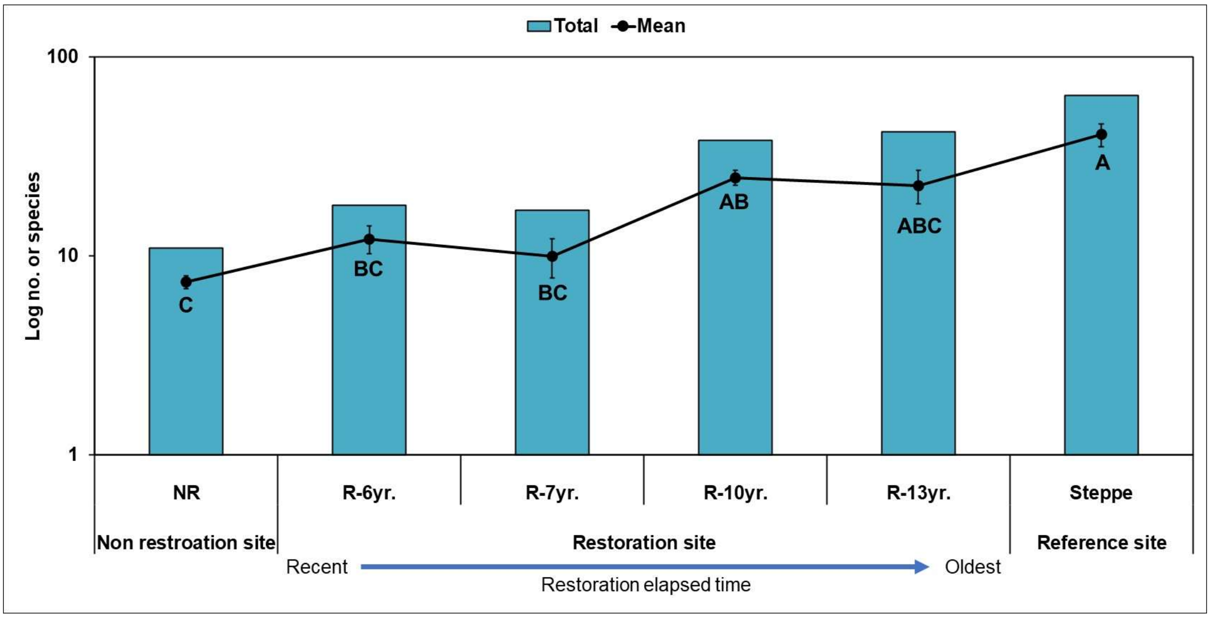

Figure S2. Comparison of plant biodiversity by research site in Hulunbuir for 5 years (2014 – 2018). Black dots represent the average number of plant species detected and error bars represent the standard deviation. Blue bars mean the total number of species present and alphabet in blue bar mean differences in mean number of species per study site. NR: site of non-restoration, R-arabic numeral yr.: site of year time after restoration. Steppe includes both temperate typical steppe (TTS) and woodland steppe (WS).

	Division	Vegetation type	Landform	Vegetation type	
Changes of vegetation due to desertifi-cation in Horqin area	Natural vegetation	Populus simonii<br>Salix matsudana<br>Salix gordejevii<br>Prunus armeniaca<br>Ulmus pumila<br>and other species	Original ecosystem	Reference sites (Steppe)<br>- TTS<br>- WS	Changes of vegetation according to the restoration in Hulunbeier
	ǀ		⟲	Γ	
	Primary change	Agropyron cristatum<br>Pennisetum flaecidum	Fixed sand dune	Agropyron cristatum<br>- R-13yr.	
	ǀ		⟲	Γ	
	Secondary change	Caragana microphylla<br>Setaria viridis	Semi-fixed sand dune	Caragana microphylla<br>- R-13yr.<br>- R-10yr.<br>- R-7yr.	
	ǀ		⟲	Γ	
	Tertiary change	Artemisia halodendron<br>Corispermum hyssopifolium<br>Salsola collina	Semi-shifted sand dune	Artemisia halodendron<br>Corispermum hyssopifolium<br>- R-10yr.<br>- R-7yr.<br>- R-6yr.	
	ǀ		⟲	Γ	
	Quarternary change	Agiophyllum arenarium<br>or none	Shifted sand dune	- NR	

Figure S3. The vegetation decline of desertification areas of Horqin (Squires et al., 2009) and process of ecosystem restoration according the vegetation restoration of Hulunbeier in Inner Mongolia.
